# Enhanced Detection of Major Pathogens and Toxins in Poultry and Livestock With Zoonotic Risks Using Nanomaterials-Based Diagnostics

**DOI:** 10.3389/fvets.2021.673718

**Published:** 2021-06-07

**Authors:** Priya K. Manhas, Irwin A. Quintela, Vivian C. H. Wu

**Affiliations:** Produce Safety and Microbiology Research Unit, U.S. Department of Agriculture, Agricultural Research Service, Western Regional Research Center, Albany, CA, United States

**Keywords:** nanomaterials, gold nanoparticles, diagnostic tools, animal diseases, zoonotic pathogens

## Abstract

Nanotechnology has gained prominence over the recent years in multiple research and application fields, including infectious diseases in healthcare, agriculture, and veterinary science. It remains an attractive and viable option for preventing, diagnosing, and treating diseases in animals and humans. The apparent efficiency of nanomaterials is due to their unique physicochemical properties and biocompatibility. With the persistence of pathogens and toxins in the poultry and livestock industries, rapid diagnostic tools are of utmost importance. Though there are many promising nanomaterials-based diagnostic tests specific to animal disease-causing agents, many have not achieved balanced sensitivity, specificity, reproducibility, and cost-effectiveness. This mini-review explores several types of nanomaterials, which provided enhancement on the sensitivity and specificity of recently reported diagnostic tools related to animal diseases. Recommendations are also provided to facilitate more targeted animal populations into the development of future diagnostic tools specifically for emerging and re-emerging animal diseases posing zoonotic risks.

## Introduction

As the world's population steadily increases, sustainable and safe agriculture remains very critical. Global trade, climate change, and modified farm practices create new opportunities for the transmission of transboundary animal diseases (1). This dynamic has resulted in foreign and endemic infectious disease outbreaks, which negatively impacted agriculture, the economy, and public health (2).

The combined production value of poultry and livestock industries in the United States amounts to almost $ 91 billion (3), but poultry and livestock disease outbreaks pose a significant challenge. A vital example of a common but fatal poultry disease is avian influenza. Highly pathogenic avian influenza (HPAI) A (H5N1) causes high fatality rates in poultry; first detected in 1996 (China) from geese, and then in humans in 1997 (Hong Kong) during a poultry outbreak. Since then, it has been under surveillance in 50 countries across four continents and remained endemic in six countries. Around 50 million birds (chickens and turkeys), which accounted for 8% of turkey meat and 12% of table-egg laying chickens in the U.S., died and/or were destroyed between 2014 and 2015 to control the spread of HPAI (4). In November 2020, 19,000 birds suspected of H5N8 infection were culled in Korea (5). From 2003 to 2020, 862 cases of human infection with H5N1 were reported globally, with 455 deaths (6). The emergence of Severe Acute Respiratory Syndrome Coronavirus 2 (SARS-CoV-2), which causes COVID-19, has infected 114 million people globally, with 2.5 million deaths as of February 2021 (7). SARS-CoV-2 and other highly genetically diversified bat-associated coronavirus strains can infect varying mammalian hosts, including bats, carnivores, pangolins, and primates (8). It is important to efficiently monitor and control these emerging and re-emerging pathogens to prevent the occurrence of outbreaks between humans and animals, that is, livestock populations.

Nanomaterials have gained prominence in diagnostics due to their combined strength and ductile properties (9). Nanoparticles such as gold nanoparticles (AuNPs) exhibit unique properties and functions on the nanodimensional scale (10, 11) and are readily visualized due to intense colors and formation of stable conjugates for highly-sensitive and specific diagnostic applications (12). This minireview aims to provide a comprehensive assessment by discussing the six emerging/re-emerging and prevalent diseases affecting poultry and livestock while focusing on both the functionalization and modifications of the integrated nanomaterials in the published diagnostic methods, balancing an optimum sensitivity and sensitivity.

## Functionalization of Nanomaterials

The inert nature of nanomaterials limits their applications; hence, it requires functionalization to allow integration into the diagnostic platforms (13). Surface functionalization and encapsulation may include small molecule ligands, polymers, and biomolecules (14). The bottom-up approach wherein nanomaterials are synthesized prior to their intended use along with their organic binders has remained popular (15). Functionalization of nanomaterials can be achieved in various approaches; however, this mini-review focuses only on those intended for immuno-based methods and molecular-based diagnostics.

## Immuno-Based Methods

Immuno-based methods utilizing nanoparticles take advantage of the antibody-antigen relationships. These diagnostic methods use antibodies as either capture/detection elements or the assay's primary targets. Antibodies conjugated into nanoparticles have proven to be excellent biorecognition elements due to stability, biocompatibility, and its sensitivity to the target antigens even at lower concentrations (16). As targets, the presence of certain antibodies in animal sera, blood, and urine can also be strong indicators of pathogen exposure and infection (17). Nanomaterials are functionalized with antigens to target these antibodies at various stages of infections (18–20). The following emerging and re-emerging veterinary infectious diseases are often diagnosed by utilizing nanomaterials in either antigen-detecting or antibody-detecting diagnostic assays.

### Avian Influenza

Avian influenza continuously assails both avian species and humans and has become a threat to health and the economy (21). Diagnostic kits for avian influenza are usually based on immunochromatography that utilizes antiviral nucleoprotein antibodies and colloidal particle-conjugated antibodies (22).

**Figure 1:**
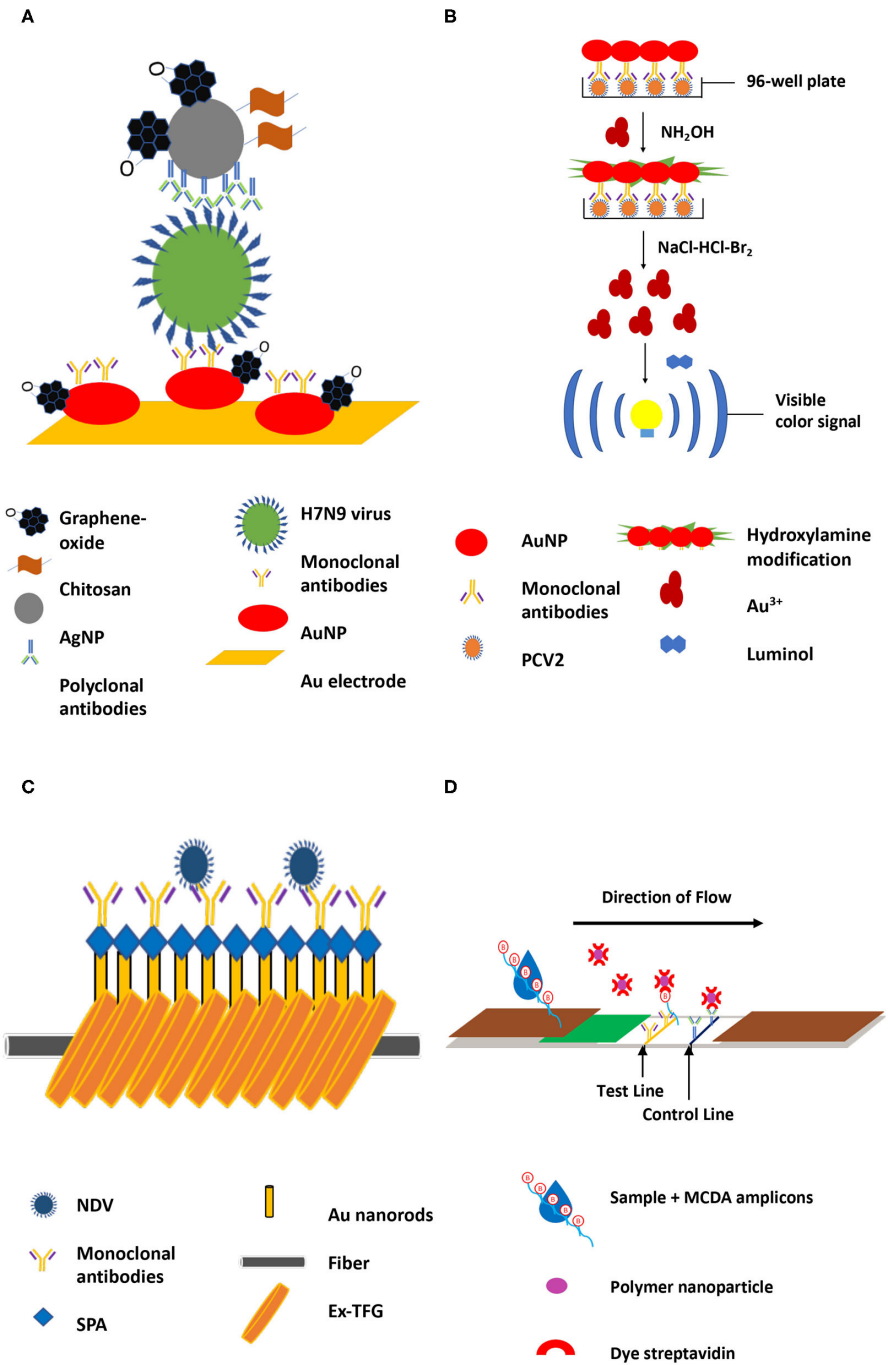
Representative animal disease diagnostic methods incorporated with various nanomaterials. **(A)**. Silver nanoparticles in immune-based electrochemical method targeting H7N9 virus (26). **(B)**. Chemiluminescence immunoassay with AuNPs targeting PCV2 (27) **(C)**. Detection of Newcastle disease through targeting ND antibodies using excessively tilted fiber grating (Ex-TFG) coated with gold nanospheres (28). **(D)**. Polymer nanoparticle-based LFB for detecting brucellosis (29).

A fluorescent immunochromatographic test strip incorporated with monoclonal antibody (MAb)-modified europium nanoparticles specific to hemagglutinin with a detection limit of 31 ng/ml was previously reported (23). Recently, a colloidal Au-based immunochromatographic strip test with two MAbs for H7N9 avian influenza viral antigen detection with a detection limit of 10^2.55^ 50% tissue culture infective dose which is equivalent to two hemagglutinin units of H7N9 (1:32 dilution) has been reported (24). Compared with PCR, this immunochromatographic strip test has a 71.4% sensitivity and 98.6% specificity (24). Similarly, an antibody-based method utilizing magnetic silica nanoparticles with a resonance light scattering system had a sensitivity range of 0.5–50 ng/ml with no cross-reactivity reaction reported (25). And finally, an immune-based electrochemical method utilizing silver nanoparticles (AgNPs) specific to H7N9, chitosan, and graphene on a gold electrode coated with AuNPs/graphene had a sensitivity of 1.6 pg/ml within 1 h while remaining highly-selective against other avian influenza strains (Figure 1A) (26). The current USDA National Veterinary Services Laboratories (NVSL) standard operating procedures for detecting avian influenza from oropharyngeal/cloacal, and fecal swab samples primarily involve real-time reverse transcriptase PCR (RT-PCR). With the integration of nanomaterials into portable diagnostic methods, pen-side testing has become a viable option without comprising sensitivity and specificity.

### Postweaning Multisystemic Wasting Syndrome

Postweaning multisystemic wasting syndrome (PMWS) is a viral infection caused by porcine circovirus type 2 (PCV2), causing wasting and pale skin, respiratory distress, and icterus in the nursery and growing pigs accompanied by a high mortality rate (30). There is no known cure for PMWS, and infected pigs have an increased rate of mortality (31). Though it is not considered as a zoonotic disease yet, PCV2 can potentially proliferate in human cells *in vitro* (32). Licensed PCV2 vaccines (Suvaxyn PC2 and Ingelvac CircoFLEX) that effectively prevent PCV2 viremia are currently available in the U.S.

Monoclonal antibodies (MAbs)-conjugated nanomaterials for PCV2 detection have been previously reported. A PCV2-specific MAb-AuNPs in a chemiluminescence immunoassay has resulted in a limit of detection (LOD) of 1.73 × 10^3^ copies/ml, which was further enhanced to 2.67 × 10^2^ copies/ml by hydroxylamine amplified AuNPs (III) (Figure 1B) (27). This assay, however, had a moderate incidence rate of cross-reactivity around 18.8% (27). A similar LOD (8 × 10^2^ copies/ml) was achieved when MAb-modified multi-branched AuNPs were used against PCV2 cap protein in surface-enhanced Raman scattering (SERS) (33). Conventional ELISA has also been modified with nanomaterials in an attempt to improve its sensitivity without increasing its cost (17, 34, 35). The horseradish peroxidase component of an enzyme-free “ELISA” has been replaced with antibody-modified gold-platinum and silica dioxide nanospheres (35). The nanospheres complex allowed a colorimetric change from red to purple with the presence of PCV2 without cross-reactivity, which resulted in a 200-fold improvement in sensitivity compared as compared to the traditional ELISA. The gold standard for the diagnosis of the disease is RT-PCR, while virus isolation, electron microscopy, and serum virus neutralization assays are also utilized; field testing is currently available. The development of nanomaterials-based assays has allowed rapid detection of PCV2 without the need for complicated and costly equipment.

### Newcastle Disease

Newcastle disease (ND) is a viral disease causing respiratory distress, lesions, cessation of egg productions, and nervous manifestations in poultry and wild avian species (36–39). Newcastle disease (ND) has been prevalent in Asia, Africa, North, and South America; the onset of infection can affect a flock within an average of 5 days post aerosol exposure. Unfortunately, ND virulent virus carries a zoonotic risk of transitory conjunctivitis to laboratory workers and other staff, that is, vaccination teams; thus, safety guidelines and protocols need to be strictly implemented and followed (40). Diagnosis often relies on viral isolation and subsequent conventional characterization, which can take 2–12 days post-exposure (41).

Similar to avian influenza, ND can also be detected and diagnosed by using immunochromatographic assays. Recently, a quantitative antigen-based Au-immunochromatographic technique incorporated with viral protein antigens/AuNPs (haemagglutinin-neuraminidase) was developed, which allowed the detection of ND viral antibodies with a sensitivity of 2^2^ titers without any cross-reactivity (42).

Fluoroimmunoassays have been also reported for ND virus detection. This method primarily relies on fluorescent signals but often lacks sensitivity. However, when magnetic separation is added as a concentration step of target analytes, sensitivity can drastically improve. Cadmium telluride quantum dots modified with NDV antibodies and mercaptosuccinic acid coupled with iron oxide nanoparticles were able to detect as low as 1.5 ng/ml of antigens without compromising specificity (43). Furthermore, an immunosensor with tilted fiber grating (Ex-TFG) coated with staphylococcal protein A-Au nanospheres was able to successfully detect NDV antibodies within 25 min (28). This method had a detection limit of 25 pg/ml, a 100-fold more sensitive than the previously reported fluoroimmunoassay (Figure 1C) (28). Furthermore, the addition of Au nanospheres has allowed a 5–10 times improvement in sensitivity compared to the Ex-TFG alone (28). Commercial ND viral diagnostic tests (i.e., IDEXX Newcastle Disease Virus Antibody Tests) are currently available such as ELISA systems that allow detection and quantification of antibodies to ND viruses. Nanomaterials will continuously improve the diagnosis and rapid testing technologies of ND viruses for effective monitoring and mitigation steps.

## Molecular-Based Methods

Nanomaterials have been modified with synthetic nucleic acid probes (i.e., aptamers and oligonucleotides) to serve as recognition elements. Molecular-based recognition elements offer significant advantages over antibodies. Antibodies have low stability at high temperatures, high production costs and variation with each subsequent batch, and a need for a constant supply of mammalian cell culture (44). In contrast, aptamers are non-immunogenic and are highly stable at various conditions maintaining their high binding affinity with the target analytes (45). The use of aptamers significantly lowers manufacturing costs compared to antibody-based techniques, as aptamers do not require complicated procedures and infrastructures such as animal care facilities (45). The following emerging veterinary infectious diseases can be detected by utilizing molecular-based diagnostic methods enhanced with nanomaterials.

### Anthrax

Anthrax is a serious infectious bacterial disease caused by *Bacillus anthracis*, which is fatal to ruminants and humans alike, and often spreads quickly through contaminated feed and water (46). In a study that examined the prevalence of *B. anthracis* spores, 30% of sheep and 27.5% of goats were positive for the presence of spores that were attached to the body of animals (47). These spores can cause a zoonotic infection in humans with a 25–60% fatality rate (48).

There are many molecular-based diagnostic techniques used to detect *B. anthracis*. One such technique used a quartz crystal microbalance (QCM) biosensor with single-stranded modified-AuNPs probes specific to *Ba813* and *pag* of *B. anthracis* and had a sensitivity of 3.5 × 10^2^ CFU/ml (49). In another study, a colorimetric assay that utilized asymmetric PCR amplicons/AuNPs complexes was able to detect *B. anthracis* at 10 pg/ml detection limit with no cross-reactivity (50). Likewise, a multi-walled carbon nanotube (MWCNTs)-based fluorescence aptasensor was reported to detect the recombinant protective antigen domain 4 (rPAD4) of *B. anthracis* within 10 min (51). The immobilized aptamer was labeled with Gel Green, and a sensitivity of 20 ng/ml and 62.5 ng/ml purified and unpurified rPAD4 protein, respectively, was reported (51). When coupled together, nanomaterials and aptamers can reduce cost as well as the turn-around time while increasing the sensitivity of the assay (50, 51). Current diagnostic tests include bacterial culture, PCR as well as ELISA for antibody detection in reference laboratories. Rapid detection using nanomaterials-based technologies will enhance the diagnosis of anthrax and assist the livestock industry in controlling the risks that it poses to animal health and the human population.

### Brucellosis

Brucellosis is a bacterial infectious disease caused by *Brucella* spp., which is endemic to ruminants causing influenza-like symptoms in both cattle and humans. It can spread quickly through cattle and humans by contaminated milk and contact with animal feces (52). *Brucella* spp. are identified by direct culture from infected tissues in selective media or direct stained smears. For serological assays, serum tube agglutination test (SAT) and milk ring test (anti-brucella antibodies) are performed.

Molecular-based methods tend to leverage the specific hybridization events between nanomaterials-based probes and target pathogens or their nucleic acids. An oligonucleotide-modified AuNP-based colorimetric assay was able to detect *Brucella* spp within 30 min by targeting the BCSP31 outer membrane protein and achieved a LOD of 10^3^ CFU/ml in bovine (urine and semen samples) and 10^4^ CFU/mL in milk with no cross-reactivity (53). Another target gene region, *IS711*, was detected using the same method with improved sensitivity of 1.09 pg/μl using unamplified *Brucella* genomic DNA (54, 55) without cross-reactivity observed. A multiple cross displacement amplification and lateral flow assay utilizing polymer nanoparticles modified with oligonucleotide probe targeting the *BSCP31* gene was able to improve the sensitivity to 10 fg (Figure 1D) (29).

### Aflatoxicosis

Aflatoxicosis is a blanket term used to describe a wide variety of symptoms indicative of poisoning caused by the metabolism of aflatoxins which are potent mycotoxins that can be present in animal feeds, grains, nuts, and animal products (56). Produced from the *Aspergillus* spp., aflatoxins come in a variety of metabolites, including aflatoxin B_1_, B_2_, G_1_, and G_2_ (57). Aflatoxin B_1_ (AFB_1_) is considered the most toxic metabolite since it is a potent carcinogen with hepatotoxic consequences in poultry, livestock, and human consumers of infected animal products (58). Many countries have strict limits of acceptable amounts of AFB_1_ in food. Specifically, the European Union sets 2 μg/kg of AFB_1_ in food while in the U.S., the maximum permitted level of AFB_1_ combined with B_2_, G_1_, and G_2_ is 20 μg/kg; 0.5 μg/kg in milk; and 100–300 μg/kg range for animal feeds (59, 60). Current detection and surveillance methods include thin-layer chromatography, high-performance liquid chromatography (HPLC), mass spectroscopy, and ELISA, among others. Nanomaterials are continuously introduced and integrated into various detection applications to improve sensitivity and reduce cost, as it is very crucial to maintain the level of aflatoxins within the allowed limits.

An aptasensor with Au nanowires/graphene oxide and aptamer was developed for AFB_1_ detection based on the differences in differential pulse voltammetry peak current (58). The method achieved a sensitivity of 1.4 pM or 0.62 ng/ml which was comparable to the gold standard of high-performance liquid chromatography (HPLC) and a shorter turn-around time of 90 min. Another study developed a molecular-based method utilizing mesoporous silica nanoparticles modified with a complex of amino groups, aptamers, and Rh6G through SERS and has an improved LOD of 0.13 ng/ml (60), which was more sensitive than other fluorescence methods.

## Future Recommendations

Nanomaterials have greatly improved the capabilities of numerous diagnostic tools, especially those designed to detect animal disease-causing pathogens and toxins. Many diagnostic tools can be operated on-site for pen-side testing or in the laboratory for initial screening of samples or rapid confirmation of some emerging and re-emerging animal diseases posing zoonotic risks. Though most diagnostic tools have improved their sensitivity in the past decade, some recognition materials are still suffering from cross-reactivity and production costs. The current immune-based diagnostic assays often fall short in sensitivity, specificity, and cost, but newer technologies, such as aptamers and other molecular biology tools can offer solutions. Nanomaterials can be functionalized in several different ways; however, this mini review only covers immune-based and molecular-based methods. This mini-review opens the door to a comprehensive risk and cost analysis when designing nanomaterials-based diagnostic tools for other novel pathogens.

## Conclusion

This minireview discusses the emerging and prevalent diseases affecting poultry and livestock and puts emphasis on both the functionalization and modifications of the integrated nanomaterials in the published diagnostic methods to provide researchers and the livestock industry with alternative solutions and approaches in the diagnosis of veterinary infectious diseases. Nanomaterials including AuNPs, AgNPs, silica nanoparticles, iron oxide nanoparticles, europium nanoparticles, cadmium telluride quantum dots, and polymer nanoparticles have become valuable in the detection of pathogens and toxins causing diseases in poultry and livestock due to their versatility and biocompatibility. Functionalization and modifications of the integrated nanomaterials in the published and reported diagnostic methods have allowed rapid detection of pathogens and toxins with superior sensitivity and specificity. However, the chosen recognition elements significantly contribute to achieving the ultimate range of detection limits; therefore, it is important to consider the advantages and disadvantages of each capture and detection element in the diagnostic systems.

## Author Contributions

PM and IQ conducted the literature review and wrote the manuscript. IQ and VW revised the manuscript, added important scientific content, and refined the interpretation of the results. VW was involved in conceptualization, funding acquisition, review and editing of the manuscript, supervision, and project administration. All the authors reviewed the final version of the manuscript and agreed to its submission.

## Conflict of Interest

The authors declare that the research was conducted in the absence of any commercial or financial relationships that could be construed as a potential conflict of interest.
